# Hyaluronan Synthase and Hyaluronidase Expression in Serous Ovarian Carcinoma is Related to Anatomic Site and Chemotherapy Exposure

**DOI:** 10.3390/ijms131012925

**Published:** 2012-10-10

**Authors:** Ilana Weiss, Claes G. Trope, Reuven Reich, Ben Davidson

**Affiliations:** 1 Institute of Drug Research, School of Pharmacy, Faculty of Medicine, The Hebrew University of Jerusalem, Jerusalem 91120, Israel; E-Mails: ilana.weiss.il@gmail.com (I.W.); c.g.trope@medisin.uio.no (C.G.T.); 2 Department of Gynecologic Oncology, Oslo University Hospital, Norwegian Radium Hospital, Oslo N-310, Norway; 3 Division of Pathology, Oslo University Hospital, Norwegian Radium Hospital, Oslo N-0424, Norway; 4 Institute of Clinical Medicine, Faculty of Medicine, University of Oslo, Oslo N-0424, Norway

**Keywords:** hyaluronan synthase, hyaluronidase, ovarian carcinoma, tumor progression, effusions, survival

## Abstract

The present study investigated the expression and clinical role of hyaluronan synthases (HAS1-3) and hyaluronidases (Hyal1-3) in serous ovarian carcinoma. *HAS* and *HYAL* mRNA expression was analyzed in 97 tumors (61 effusions, 27 primary carcinomas, 9 solid metastases) using PCR and further studied for association with clinicopathologic parameters, including survival. *HAS1* mRNA was overexpressed in effusions compared to primary carcinomas and solid metastases (*p* < 0.001), and an alternatively spliced *HAS1* was expressed only in effusions. *HAS2* mRNA was overexpressed in solid metastases and primary carcinomas compared to effusions (*p =* 0.043), and *HAS3* mRNA was overexpressed in primary carcinomas and effusions compared to solid metastases (*p =* 0.008). *HYAL1* mRNA was absent in all specimens, whereas *HYAL2* was expressed as two splice variants, of which *HYAL2*-var2 was overexpressed in solid metastases compared to effusions and primary carcinomas (*p* < 0.001). *HYAL3* mRNA was expressed as wild-type and variant 1–3 form, the latter more highly in primary carcinomas and effusions compared to solid metastases (*p =* 0.006). *HAS1* mRNA was overexpressed in pre- compared to post-chemotherapy effusions (*p* < 0.001), with opposite finding for *HYAL2*-var1 and *HYAL3*-WT (*p =* 0.016 and *p =* 0.024, respectively). Higher *HYAL2*-var1 and *HAS1* splice variant mRNA expression in effusions was associated with longer (*p =* 0.033) and shorter (*p =* 0.047) overall survival, respectively. These data are the first to document a role for HAS and Hyal members in tumor progression in ovarian carcinoma, as evidenced by their differential expression as function of anatomic site and chemotherapy exposure, with a possible prognostic role for patients with malignant effusions.

## 1. Introduction

Ovarian carcinoma (OC) is the most lethal gynecologic malignancy. The majority of patients are diagnosed with advanced-stage disease and despite aggressive surgery and adjuvant platinum-based combination therapy, succumb to their disease, primarily due to chemotherapy resistance [1,2]. Despite the obvious role of FIGO stage, tumor grade, residual disease (RD) volume after surgery and the presence of ascites in providing prognostic information in OC, these factors fail to predict clinical outcome for individual patients owing to a significant degree of tumor heterogeneity. Widely accepted biological factors that are able to predict chemoresponse and survival in OC are unavailable to date, a fact that in part reflects our limited understanding of metastatic OC, the cause of death for most patients.

The extracellular matrix (ECM) is a critical environmental determinant of tumor cell behavior. The matrix serves as a scaffold for the tumor cells to adhere, migrate and proliferate in response to the reservoir of growth factors and cytokines stored in it. Hyaluronan (also termed hyaluronic acid or hyaluronate; HA) is a prominent component of the ECM in many tissues, particularly in rapidly remodeling ones. Hyaluronan is a very large, linear, negatively-charged polysaccharide, which is composed of repeating non-sulphated disaccharides of glucuronate and *N*-acetylglucosamine and exists as a high molecular weight polymer of approximately 10^4^–10^7^ Da. Its strong negative charge attracts a large associated volume of water capable of expansion up to 10,000 times its actual polymer volume. HA functions in normal physiology by maintaining tissue hydration and osmotic balance in tissues, and additionally regulates cell adhesion, migration, apoptosis and proliferation via interaction with specific cell surface receptors. In addition to its role in embryogenesis, there is increasing evidence for the involvement of HA in tumor progression of multiple cancers, through its effect on proliferation, apoptosis, angiogenesis, migration and invasion, as well as promotion of epithelial-to-mesenchymal transition (EMT) [3,4]. Multiple studies have documented the role of HA and its receptor CD44 in the binding of OC cells to mesothelial cells, a critical event in OC dissemination in the abdomen, and its role in tumor progression and in determining prognosis in this cancer [5].

The biosynthesis of HA is regulated by three HA synthase isozymes, HAS1-3. There is a 50%–70% amino acid sequence identity between the different genes. However, they differ in their enzymatic activities. HAS1 and HAS2 synthesize HA molecules with high molecular weight, whereas HAS3 is produces low molecular weight molecules. HAS2 is the most commonly expressed family member in normal mammalian tissues, whereas HAS3 is the predominant enzyme in pathological conditions, such as inflammation and cancer [6].

HA degradation is induced by a family of six hyaluronidases, consisting of Hyal1, Hyal2, Hyal3, Hyal4, PH-20/PSAM1 and the pseudogene HyalP1 [7]. Of these, Hyal1 and Hyal2 are the most widely distributed and best characterized. The major transcript of Hyal3 does not possess enzymatic activity by itself and may merely have a supportive role in Hyal1 expression [8].

Despite the wealth of literature regarding HA and CD44 expression in OC, little is known regarding the expression and clinical role of HA-synthesizing and HA-metabolizing enzymes in OC. Hyaluronidase activity was reported to be high in malignant ovarian tumors compared to their endometrial and cervical counterparts [9]. However, hyaluronidase activity was lower in OC compared to benign and borderline tumors of the ovary in another study [10]. In a study of HAS1-3 expression in 33 primary OC, HAS1 expression was reported to be associated with shorter overall survival (OS) [11]. In the only study in which both HAS and Hyal members were investigated, analysis of 39 ovary specimens, including 20 serous OC, demonstrated expression of HAS2 and HAS3 mRNA, with low or undetectable HAS1, and HAS mRNA levels were not significantly related to HAS protein or hyaluronan accumulation. HYAL1 mRNA level was lower in grade 3 OC compared to normal ovaries and correlated with the enzymatic activity of tissue hyaluronidases, and inversely with HA staining intensity, with no such association for HYAL2 [8].

Only one of the above studies analyzed metastatic OC specimens, and none included effusion specimens. Metastatic OC, particularly effusions, have different expression of cancer-associated molecules at the mRNA and protein level [12]. OC of different histology is additionally increasingly regarded to represent different diseases with a unique biology and expression profile [13,14]. In view of these observations, the present study focused on the expression and clinical role of the various HAS and Hyal enzymes and their splice variants in serous OC effusions, and compared these expression pattern with solid primary and metastatic serous OC specimens.

## 2. Results

### 2.1. HAS and HYAL Expression is Significantly Different at Various Anatomic Sites in OC

**Figure 1:**
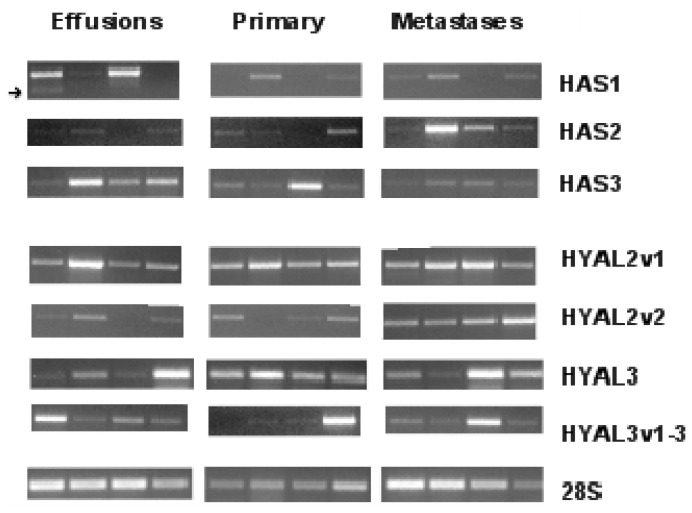
Hyaluronan synthases (*HAS*) and hyaluronidases (*HYAL*) mRNA in serous ovarian carcinoma: Examples of *HAS* and *HYAL* mRNA expression in effusions, primary carcinomas and solid metastases from patients with serous ovarian carcinoma. The *28S* RNA served as control. *HYAL2* levels were analyzed using quantitative PCR (qPCR), but a gel was nevertheless run in addition, in order to be able to visually present all enzymes similarly.

**Figure 2:**
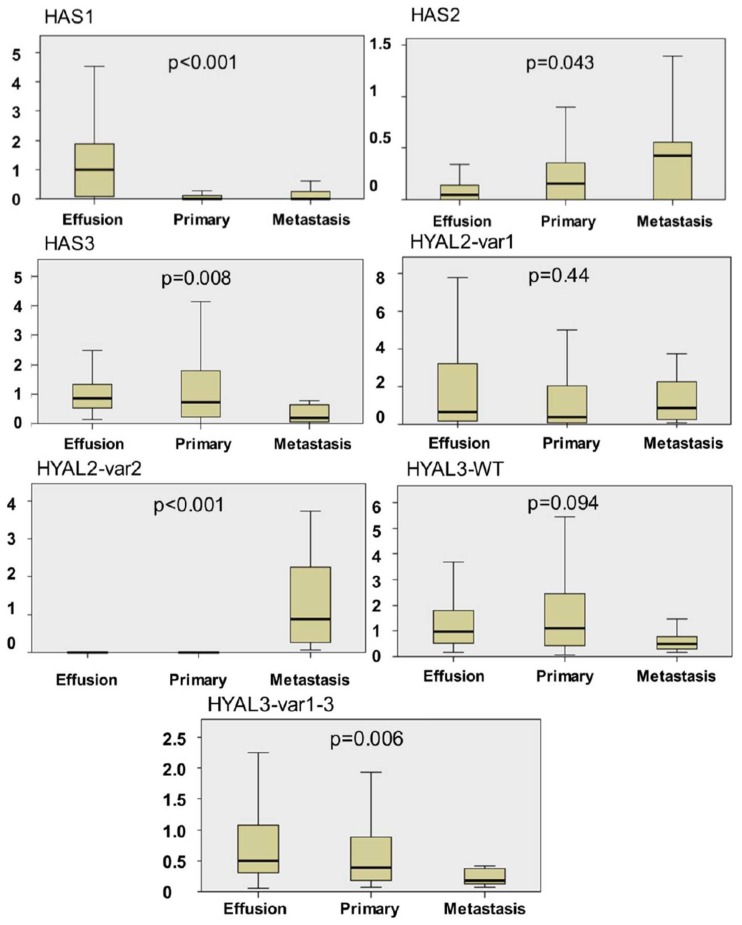
Anatomic site-related differences in *HAS* and *HYAL* mRNA expression. Graphic illustration of the differences in *HAS1-3* and *HYAL2* mRNA expression between ovarian carcinoma effusions, primary ovarian carcinomas and solid metastases. *HAS1* mRNA expression is significantly higher expression in effusions compared to the two other anatomic sites (*p* < 0.001); *HAS2* mRNA levels are higher in solid metastases and primary carcinomas compared to effusions (*p =* 0.043); *HAS3* mRNA expression levels are significantly higher in primary carcinomas and effusions compared to solid metastases (*p =* 0.008); *HYAL2*-var1 expression is comparable in effusions, primary carcinomas and solid metastases (*p =* 0.44); *HYAL2*-var2 expression is significantly higher in solid metastases compared to effusions and primary carcinomas (*p* < 0.001); *HYAL3*-WT expression is higher in primary carcinomas and effusions compared to solid metastases, though not significantly (*p =* 0.094). *HYAL3*-var1-3 expression is significantly higher in primary carcinomas and effusions compared to solid metastases (*p =* 0.006). The bars on the *Y*-axis represent arbitrary units.

HAS1 mRNA was detected in 47/61 (77%) effusions, 8/27 (30%) primary OC and 4/9 (44%) solid metastases, with a comparative analysis of expression levels showing significantly higher expression in effusions compared to the two other anatomic sites (*p* < 0.001) (Figures 1 and 2). We additionally observed that HAS1 is alternatively spliced in tumor cells derived from effusions, but not in solid specimens. The splice variant has a deletion at exon 4, and was found in 22 effusions (Figure 1).

HAS2 mRNA was found in 30/61 (49%) effusions, 18/27 (67%) primary OC and 5/9 (56%) solid metastases, with expression levels being moderately higher in solid metastases and primary OC compared to effusions (*p =* 0.043) (Figures 1 and 2).

HAS3 mRNA was detected in 61/61 (100%) effusions, 26/27 (96%) primary OC and 8/9 (89%) solid metastases, with a comparative analysis of expression levels showing significantly higher expression in primary OC and effusions compared to solid metastases (*p =* 0.008) (Figures 1 and 2).

HYAL1 mRNA was not detected in any of the specimens analyzed, irrespective of the anatomic site (Figure S1).

HYAL2 is alternatively spliced and has two variants. The two variants produce similar protein products but differ in the first exon. HYAL2-var1 was found in all specimens except for 3 effusions, with comparable levels at the 3 anatomic sites (*p =* 0.44). In contrast, HYAL2-var2 was detected in 14/51 (27%) effusions, 4/25 (16%) primary OC and 9/9 (100%) solid metastases, with significantly higher expression in solid metastases compared to effusions and primary carcinomas (*p* < 0.001) (Figures 1 and 2).

HYAL3 is also alternatively spliced and has 4 variants. However, only the wild-type (WT) form encodes for an active protein [15]. Due to the unique structure of the gene, we could not generate probes that selectively recognize the four variants. Our primers recognized the WT form and var1-3 collectively. Both HYAL forms were found in all specimens at the 3 anatomic sites. However, expression levels were higher in primary carcinomas and effusions compared to solid metastases, this difference being a trend for HYAL3-WT (*p =* 0.099) and significant for HYAL3-var1-3 (*p =* 0.006) (Figures 1 and 2).

### 2.2. HAS and HYAL Expression Is Significantly Related to Clinicopathologic Parameters in OC Effusions

Clinicopathologic data of effusion cohort are detailed in Table 1. HAS and HYAL expression in effusions was studied for potential correlation with clinicopathologic parameters. The main differences observed were related to chemotherapy exposure. HAS1 mRNA levels were significantly higher in pre-compared to post-chemotherapy effusions (*p* < 0.001), and the same difference was observed for its splice variant (*p* = 0.022). In contrast, HYAL2-var1 and HYAL3-WT levels were higher in post-chemotherapy effusions (*p* = 0.016 and *p* = 0.024, respectively). The differences in HAS1 expression was significantly related to previous exposure to platinum (*p* < 0.001), whereas both HAS1 and HYAL3-WT levels were related to exposure to paclitaxel (*p* = 0.003 and *p* = 0.005, respectively).

HAS and HYAL expression was unrelated to effusion site, patient age, FIGO stage, RD volume or response to chemotherapy at diagnosis (*p* > 0.05). Data are summarized in Table 2.

The follow-up period for the 56 OC patients with effusions ranged from 2 to 81 months (mean = 36 months, median = 33 months). PFS ranged from 0 to 40 months (mean = 8 months, median = 6 months), with 12 patients never achieving a disease-free period. At the last follow-up, all patients had died of the disease.

**Figure 3:**
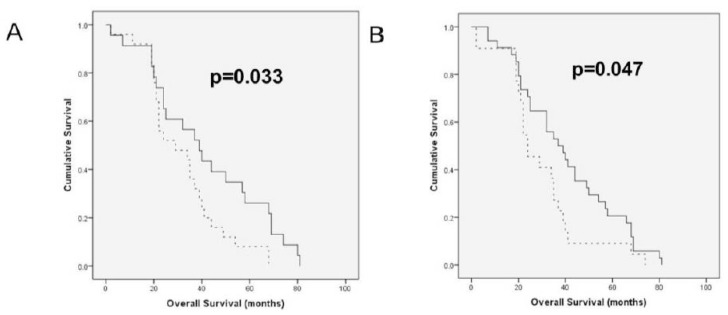
*HAS* and *HYAL* mRNA expression in serous ovarian carcinoma effusions correlates with overall survival (OS) (**A**) Kaplan-Meier survival curve showing the association between *HYAL2*-var1 mRNA levels in effusions and OS for 48 patients. Patients with effusions showing low (below median) *HYAL2*-var1 expression (*n* = 25, dashed line) had a mean OS of 32 months compared to 42 months for patients with tumors with high *HYAL2*-var1 expression (*n* = 23, solid line; *p =* 0.033). (**B**) Kaplan-Meier curve showing the association between the presence of the *HAS1* splice variant in effusions and OS for 56 patients. Patients with effusions expressing the splice variant (*n* = 22, dashed line) had a mean OS of 29 months compared to 40 months for patients with tumors negative for the variant (*n* = 34, solid line; *p* = 0.047).

In survival analysis of the entire cohort, higher HYAL2-var1 mRNA expression was associated with longer OS (*p =* 0.033; Figure 3A), whereas higher HAS1 splice variant expression was significantly related to shorter OS (*p =* 0.047; Figure 3B). The survival analyses without significant associations are presented in Supplementary Figure 2. None of the studied molecules was significantly related to PFS.

The clinical parameters studied for association with survival were patient age, histological grade, FIGO stage and residual disease volume. None of these had prognostic value in this cohort, FIGO stage being the only one with *p*-value < 0.2 (*p* = 0.076).

In Cox survival analysis including HYAL2-var1 mRNA, the HAS1 splice variant and FIGO stage, none of these parameters was an independent predictor of OS.

## 3. Discussion

HA is widely expressed in human cancer and has been shown to have a central role in tumor biology [3]. Recently, blocking of HA by small oligosaccharides [16] and binding of paclitaxel to HA in order to improve tumor-specific delivery [17–19] have been investigated as therapeutic approaches in OC models *in vivo*. This emphasizes the need to study HA and its regulators in primary and metastatic disease. HA levels in tissues are affected by the balance between the activities of HAS and Hyal enzymes. In the present study, we analyzed the dynamics in the expression of these enzymes at the various anatomic sites affected by OC. We limited our cohort to patients with serous OC, the majority of which, including all patients with effusions, were diagnosed with advanced-stage disease, in order to avoid confounding factors, and primarily the different expression patterns in OC of different histological type.

Hiltunen and co-workers previously showed higher HA levels in OC metastases compared to primary tumors, as well as benign and borderline tumors of the ovary, whereas hyaluronidase activity was lower in malignant compared to benign and borderline tumors [10]. Higher levels of stromal HA were reported in another study by the same group [20]. In our series, effusions had significantly higher HAS1 mRNA expression compared to primary OC and solid metastases, and were the only site were the alternative HAS1 variant was expressed, while the opposite was true for HAS2. HAS3 was more highly expressed in primary OC and effusions compared to solid metastases. The higher expression of HAS1 in effusions concurs with some extent with the observation of Nykopp *et al.* [8], who found very low levels of this enzyme in primary serous OC, although our series had more easily detectable expression of this enzyme also in primary OC.

Similarly to HAS members, Hyal family members were differently expressed when the 3 anatomic sites studied were compared. HYAL2-var2 mRNA was more highly expressed in solid metastases compared to effusions and primary carcinomas, whereas the opposite was true for HYAL3-var1-3. HYAL1 was uniformly absent. The latter finding is in agreement with the data Nykopp *et al*., who observed reduced HYAL1 mRNA expression in OC compared to normal ovaries, benign tumors and borderline tumors using qRT-PCR [8].

Our data suggest that HAS and Hyal family members, with the exception of Hyal1, are expressed at all anatomic sites in serous OC, but with changing expression levels. Although the majority of specimens were not patient-matched, this finding suggests a dynamic HAS synthesis along tumor progression in OC. The biological role of the splice variants we detected for HAS1, HYAL2 and HYAL3 in OC is yet to be studied.

Based on the differences in HAS and Hyal expression at the different anatomic sites, it seems to us that the more advanced disease is associated with a switch in the size of the synthesized HA to a shorter form and accompanied with an increase the catabolic power.

To the best of our knowledge, this study is the first to compare pre- and post-chemotherapy OC. The levels of HAS1 mRNA and its splice variant were significantly higher in pre-chemotherapy compared to post-chemotherapy effusions, whereas HYAL2-var1 and HYAL3-WT levels were significantly higher in post-chemotherapy effusions. The differences in HAS1 expression was significantly related to previous exposure to platinum, whereas both HAS1 and HYAL3-WT levels were related to exposure to paclitaxel. This suggests a change in the equilibrium between HA synthesis and catabolism following chemotherapy, which, in the absence of stromal cells in effusions, may reflect altered tumor phenotype through selection of chemoresistant tumor clones or direct chemotherapy effect. This finding should ideally be confirmed in analysis of patient-matched pre- and post-chemotherapy specimens, although the latter are not ubiquitous in most centers.

Stromal HA expression in primary OC was reported to be associated with clinicopathologic parameters of aggressive disease, as well as poor PFS and OS, the latter finding retaining an independent prognostic role in multivariate analysis [20]. In the only analysis of the prognostic role of HAS members to date, HAS1 protein expression by immunohistochemistry in a series of 33 primary OC was related to significantly shorter survival, whereas HAS2 and HAS3 had no prognostic role. In agreement with this report, higher HAS1 splice variant expression in OC effusions was significantly related to shorter OS. This suggests that the clinical relevance of HAS1 is retained along disease progression. In another view of the inverse relationship between HAS and Hyal members, higher HYAL2-var1 mRNA expression in effusions was associated with longer OS. Although these findings did not retain its significance in Cox analysis, a larger study may yet identify these molecules as independent prognostic markers. The association of HAS and Hyal members with shorter and longer OS, respectively, suggests that HA synthesis is associated with more aggressive disease in metastatic OC, with opposite results for HA breakdown, well in agreement with the role of HA in metastasis and tumor progression in OC and in other cancers.

## 4. Materials and Methods

### 4.1. Patients and Material

A total of 97 specimens from patients diagnosed with ovarian, peritoneal or tubal serous carcinoma were obtained from the Department of Gynecological Oncology at the Norwegian Radium Hospital from 1998–2003. Due to their closely linked histogenesis and phenotype, all are referred to as OC henceforth. Informed consent was obtained according to national guidelines. The study was approved by the Regional Committee for Medical Research Ethics in Norway.

Effusions: Sixty-one fresh non-fixed malignant peritoneal (*n* = 43) and pleural (*n* = 18) effusions were obtained from 56 patients (3 patients with 2 effusions, 1 with 3 effusions). Forty-seven patients had OC, 7 had primary peritoneal carcinoma, and 2 had tubal carcinoma. The majority of patients (48/56; 86%) received platinum-based therapy, most frequently (42 patients) in combination with paclitaxel.

Effusions were submitted for routine diagnostic purposes and were processed immediately after tapping. Cell blocks were prepared using the Thrombin clot method. Diagnoses were established using morphology and immunohistochemistry. Effusion specimens were centrifuged immediately after tapping, and cell pellets were frozen at −70 °C in equal amounts of RPMI 1640 medium (GIBCO-Invitrogen, Carlsbad CA) containing 50% fetal calf serum (PAA Laboratories GmbH, Pasching, Austria) and 20% dimethylsulfoxide (Merck KGaA, Darmstadt, Germany). Smears and H&E-stained cell block sections were reviewed by a surgical pathologist experienced in cytopathology (BD). The tumor cell population was >50% in all specimens.

Biopsies: Thirty-six solid specimens (27 primary serous carcinomas, 9 metastases) were studied for comparative purposes. Metastases were from the omentum (*n* = 7), uterus (*n* = 1) or lymph node (=1). Specimens were not patient-matched, with the exception of one paired effusion and primary carcinoma, one paired effusion and solid metastases, and 2 metastases from a third patient (omentum and lymph node).

Clinicopathologic data were available for 25 of the 27 patients with primary OC. Histological grade was as follows: low-grade: 4 tumors; high-grade: 21 tumors. One patient was diagnosed with FIGO stage II disease, 18 at stage III and 6 at stage IV. Twenty-two tumors were obtained prior to chemotherapy exposure, and 3 were obtained after neoadjuvant chemotherapy. Data regarding residual disease volume were available for 23 patients, of whom 8 were debulked to 0 cm macroscopic disease, 6 to 1 cm and 9 to ≥ 2 cm.

Surgical specimens were submitted for routine diagnostic purposes, snap-frozen and kept at −70 °C with no medium. Frozen sections from all tumors were evaluated for the presence of a >50% tumor component and absence of necrosis. H&E-stained sections were reviewed by a gynecological pathologist (BD) to establish tumor type and histological grade.

### 4.2. RT-PCR Analysis

Total RNA was extracted using a commercial kit (Tri Reagent; Sigma-Aldrich, St. Louis, MO, USA), and 0.5 μg total RNA were reverse-transcribed using M-MLV Reverse Transcriptase (Promega, Madison, WI, USA) with incubation for 2 h at 37 °C, followed by 5 min at 95 °C, and diluted to 1:5 with RNase-free water. The RT-PCR analysis was performed on cDNA samples on a DNA thermal cycler (Eppendorf Mastercycler gradient; Eppendorf, Hamburg, Germany) using primer sets detecting HAS and HYAL transcripts and 28S ribosomal RNA. Primer sequences and cycle parameters are detailed in Tables 3 and 4, respectively. RNA from HT-1080 fibrosarcoma cells served as control.

Products were separated on 1.5% agarose gels, isolated using the Invisorb^®^ Spin DNA extraction kit (Invitek GmbH, Berlin, Germany) and sequenced. Gels were photographed by the KODAK EDAS 290 system. Densitometer analysis of films was performed using a computerized image analysis (NIH IMAGE 1.63) program. mRNA levels were established by calculating the target molecule/28S ratio (all cases scored for band intensity compared to control). Expression intensity of ≤5% of control levels was interpreted as negative. Measurements were made at the linear phase of the reaction.

### 4.3. Quantitative PCR (qPCR)

mRNA levels of HYAL2, 28S and the reference gene RPLPO were analyzed in 84 specimens (52 effusions, 25 primary OC, 7 solid metastases) by qRT-PCR. cDNA was amplified by qPCR in 18 μL reactions using the DyNAmo™ SYBR^®^ Green qPCR Kit with ROX™ passive reference dye (Finnzymes Oy, Espoo, Finland), with 200 nM of sense and antisense primers. The cycling program was according to the manufacturer’s instructions. The reaction was performed on the Mx3000P^®^ QPCR System (Stratagene). Oligonucleotide primers were designed by the Primer Express program (Applied Biosystems, Foster City, CA, USA). Primer sequence and assay conditions are detailed in Tables 5 and 6, respectively.

### 4.4. Statistical Analysis

Data were analyzed using the SPSS-PC package, version 18.0 (Chicago, IL, USA). Comparative analysis of HAS and HYAL mRNA expression level in effusions, primary carcinomas and solid metastases were performed using the Kruskal-Wallis H test. Analyses of the association between expression levels and clinicopathologic parameters for patients with effusions were performed using the Mann-Whitney U test. For this analysis, clinicopathologic parameters were grouped as follows: Age: ≤60 *vs.* >60 years; effusion site: peritoneal *vs.* pleural; FIGO stage: III *vs.* IV; chemotherapy status: pre- *vs.* post-chemotherapy specimens; RD volume (≤1 cm *vs.* >1 cm); response to chemotherapy for primary disease and for disease recurrence: complete *vs.* partial response/stable disease/progression. Histological grade was not included in these analyses as the majority of patients had high-grade tumors.

Progression-free survival (PFS) and OS were calculated from the date of the last chemotherapy treatment/diagnosis to the date of recurrence/death or last follow-up, respectively. Univariate survival analyses of PFS and OS were executed using the Kaplan-Meier method and log-rank test. Expression categories in survival analyses were clustered as higher *vs.* lower than median expression. Multivariate analysis of OS was performed using the Cox proportional hazard model (Method = Enter).

## 5. Conclusion

We present the first comprehensive characterization of HAS and Hyal members in metastatic OC, as well as the first study investigating the association between these molecules and chemotherapy exposure in clinical OC. Our data suggest altered expression of enzymes from both families as a function of the anatomic site and previous exposure to chemotherapy, as well as opposing prognostic roles in effusion specimens. The association of HAS1 with more aggressive disease suggests that targeting HA synthesis in OC may be a relevant therapeutic approach.

## Supplementary Materials



## Figures and Tables

**Table 1:** Clinicopathologic data of the effusion cohort (56 patients).

Parameter		

Age	Mean (range)	Number of patients 61 (38–79)
FIGO stage	III	28
	IV	28

Grade	Low	4
	High	46
	NA a	6

Residual disease	≤1 cm	20
	>1 cm	28
	NA b	8

Chemotherapy status c	Pre-chemotherapy	30
	Post-chemotherapy	31

Chemotherapy response at diagnosis	Complete	33
	Non-complete d	16
	Other e	7

a NA means not available, including effusions from inoperable patients where biopsy was too small for grading or patients were operated on in other hospitals, for which the primary tumor could not be accessed for assessment of grade;

b NA means not available, including patients operated on in other hospitals and patients with no record;

c For 61 effusions;

d Partial response, stable disease or progression;

e Allergic or adverse reaction or non-measurable response.

**Table 2:** The association between clinicopathologic data of the effusion cohort and HAS/HYAL expression (*p*-value).

Clinical Parameter	Enzyme						

	*HAS1*	*HAS2*	*HAS3*	*HYAL2v1*	*HYAL2v2*	*HYAL3wt*	*HYAL3v1-3*
Effusion site	0.4	0.28	0.76	0.23	0.97	0.9	0.37
Age	0.71	0.23	0.68	0.84	0.18	0.4	0.89
FIGO stage	0.06	0.15	0.62	0.61	0.77	0.8	0.54
RD volume	0.78	0.54	0.31	0.15	0.87	0.19	0.54
Chemoresponse	0.99	0.59	0.3	0.94	0.23	0.35	0.06
Previous chemotherapy	<0.001	0.23	0.5	0.016	0.79	0.024	0.34
Previous platinum	<0.001	0.4	0.83	0.17	0.71	0.06	0.82
Previous paclitaxel	0.003	0.24	0.72	0.43	0.58	0.005	0.54

**Table 3:** Primers for RT-PCR.

mRNA	Primers	Product size (bp)
***HAS1***	Sense: 5′-GAGGCCTGGTACAACCAGAA-35′	551
	Antisense: 55′-GCAGAGGGACGTAGTTAGCG-35′	
***HAS2***	Sense: 55′-AAGGCTAACCTACCCTGGGA-35′	523
	Antisense: 55′-AATGCACTGAACACACCCAA-35′	
***HAS3***	Sense: 55′-GACGACAGCCCTGCGTGT-35′	342
	Antisense: 55′-TTGAGGTCAGGGAAGGAGAT-35′	
***HYAL1*** **wt + variant 1–4**	Sense: 55′-GGTCAGGAAATTTGGAGGAT-35′	1154
	Antisense: 55′-ACAGGGCTTGACTGCAGAGA-35′	
***HYAL1*** **variant 5**	Sense: 55′-GTGGACAAAGAACACTCCCT-35′	1210
	Antisense: 55′-ACAGGGCTTGACTGCAGAGA-35′	
***HYAL3***	Sense: 55′-ACACACCGGAGATCTGGGAG-35′	100 + 200
	Antisense: 55′-CTGGTCACATTGATCACATA-35′	
***28S***	Sense: 55′-GTTCACCCACTAATAGGGAACGTGA-35′	212
	Antisense: 55′-GGATTCTGACTTAGAGGCGTTCAGT-35′	

**Table 4:** RT-PCR conditions.

Gene	Heating	Denaturation	Annealing	Extension	Cycles
***HAS1***	94 °C-5 min	94 °C-15 s	62 °C-30 s	72 °C-20 s	35
***HAS2***	94 °C-5 min	94 °C-15 s	60 °C-30 s	72 °C-20 s	32
***HAS3***	94 °C-5 min	94 °C-15 s	58 °C-30 s	72 °C-20 s	35
***HYAL1***	94 °C-5 min	94 °C-15 s	59 °C-30 s	72 °C-40 s	40
***HYAL3***	94 °C-5 min	94 °C-15 s	55.7 °C-30 s	72 °C-20 s	33
***28S***	94 °C-5 min	94 °C-15 s	63 °C-20 s	72 °C-10 s	16

**Table 5:** Primers for quantitative PCR (qPCR).

mRNA	Primers	Product size (bp)
***HYAL2*** **var1**	Sense: 55′-GGACTCCCACACAGTTCCTG-35′	157
	Antisense: 55′-GTGAAGATGGGTGGTGCTGT-35′	
***HYAL2*** **var2**	Sense: 55′-GCGCGAGTTCCTGAGCTG-35′	154
	Antisense: 55′-CAGTGAAGATGGGTGGTGCT-35′	
***28S***	Sense: 55′-GTTCACCCACTAATAGGGAACGTGA-35′	212
	Antisense: 55′-GGATTCTGACTTAGAGGCGTTCAGT-35′	
***RPLPO***	Sense: 55′-CCAACTACTTCCTTAAGATCATCCAACTA-35′	108
	Antisense: 55′-ACATGCGGATCTGCTGCA-35′	

**Table 6:** qPCR conditions.

Gene	Heating	Denaturation	Annealing	Extension	Cycles
***HYAL2***	95 °C-7 min	95 °C-30 s	63 °C-1 min	72 °C-30 s	40
***28S***	95 °C-7 min	95 °C-30 s	60 °C-1 min	72 °C-30 s	40
***RPLPO***	95 °C-7 min	95 °C-30 s	60 °C-1 min	72 °C-30 s	40
