# Supplementary material for: Hyaluronan Synthase and Hyaluronidase Expression in Serous Ovarian Carcinoma is Related to Anatomic Site and Chemotherapy Exposure
Source: Int J Mol Sci. 2012 Oct 10;13(10):12925–38. doi: 10.3390/ijms131012925 (PMC3497304; doi:10.3390/ijms131012925)
Supplement: Supplementary file 1 [file ijms-13-12925-s001.pdf]

Supplementary Information

Figure S1. *HYAL1* mRNA expression. Nine ovarian carcinomas (3 effusions, 3 primary carcinomas, 3 metastases, are all negative. C = HT-1080 cells.

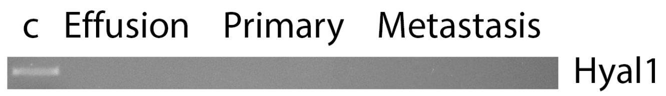

Figure S2. Survival curves for HAS2, HAS3, HYAL2v2, HYAL3v1-3 and HYAL3wt mRNA expression in effusions. None is significantly associated with overall survival.

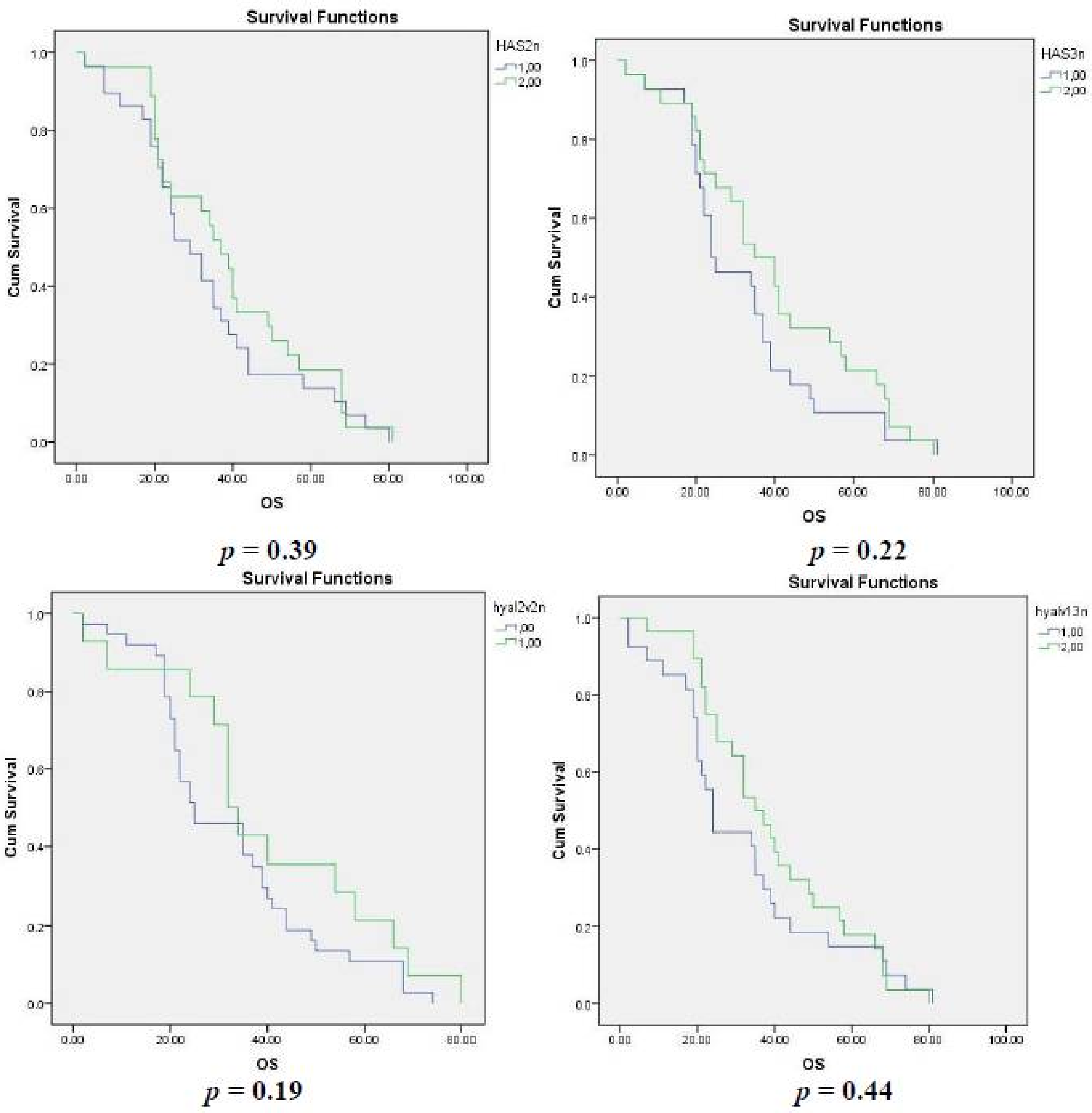

Figure S2. *Cont.*

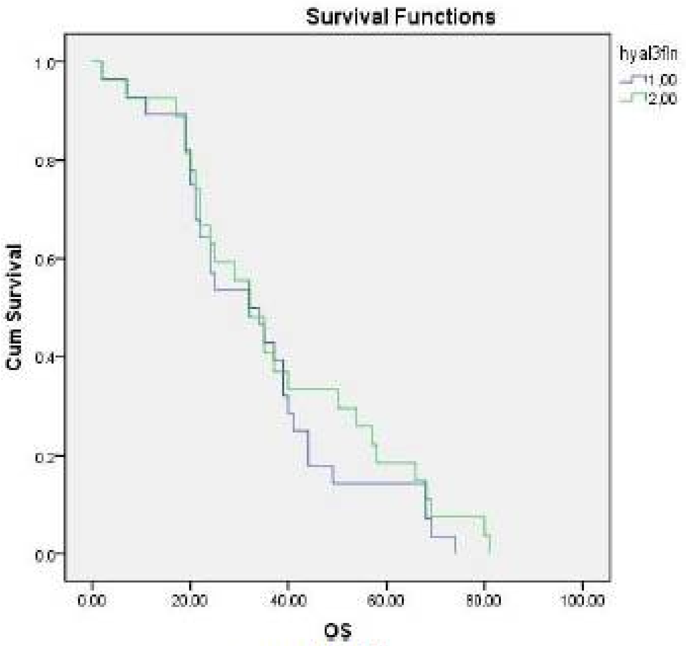

$p = 0.44$

© 2012 by the authors; licensee MDPI, Basel, Switzerland. This article is an open access article distributed under the terms and conditions of the Creative Commons Attribution license (http://creativecommons.org/licenses/by/3.0/).
